# Association between anxiety, depression symptoms, and academic burnout among Chinese students: the mediating role of resilience and self-efficacy

**DOI:** 10.1186/s40359-024-01823-5

**Published:** 2024-06-07

**Authors:** Wanqing Liu, Ruiyun Zhang, Huan Wang, Andrew Rule, Min Wang, Cody Abbey, Manpreet K. Singh, Scott Rozelle, Xinshu She, Lian Tong

**Affiliations:** 1 Department of Maternal, Child and Adolescent Health, School of Public Health, Key Laboratory of Public Health Safety, Fudan University, Ministry of Education of China, 138 Yixueyuan Road, P.O. Box 244, Shanghai, 200032 China; 2 Stanford Center of China’s Economy and Institutions, Freeman Spogli Institute of International Studies, Stanford University, Stanford, Palo Alto, CA 94305 USA; 3 University of California, Davis, CA 95616 USA; 4 Stanford University School of Medicine, Stanford, Palo Alto, CA 94305 USA; 5 Department of Pediatrics, Stanford University School of Medicine, 453 Quarry Road, Mail Code 5660, Stanford, Palo Alto, CA 94305 USA

**Keywords:** Academic burnout, Anxiety, Depression, Resilience, Self-efficacy, Children and adolescent

## Abstract

**Background:**

To explore the associations between anxiety and depression symptoms and academic burnout among children and adolescents in China, and to examine the role of resilience and self-efficacy in addressing academic burnout.

**Methods:**

A total of 2,070 students in grades 4–8 were recruited from two primary and three middle schools in Shanghai, completed the Elementary School Student Burnout Scale (ESSBS), the Multidimensional Anxiety Scale for Children-Chinese (MASC-C), the Center for Epidemiological Studies Depression Scale (CES-D), the Connor-Davidson Resilience Scale (CD-RISC), and the General Self-Efficacy Scale (GSES), with 95.04% effective response rate. Multivariable regression analyses examining the associations between anxiety / depression symptoms and academic burnout (as well as the associations between resilience / self-efficacy and academic burnout) were performed using STATA 16.0 and SmartPLS 3.0.

**Results:**

Anxiety symptoms (*β* = 0.124, *p* < 0.01) and depression symptoms (*β* = 0.477, *p* < 0.01) were positively correlated with academic burnout. Resilience partially mediated the association between depression symptoms and academic burnout (*β* = 0.059, *p* < 0.01), with a mediation rate of 12.37%. Self-efficacy partially mediated the associations between anxiety symptoms and academic burnout (*β* = 0.022, *p* < 0.01) and between depression symptoms and academic burnout (*β* = 0.017, *p* < 0.01), with mediation rates of 17.74% and 3.56%, respectively. Resilience and self-efficacy together (*β* = 0.041, *p* < 0.01) formed a mediating chain between depression symptoms and academic burnout, with a mediation rate of 8.6%.

**Conclusions:**

Anxiety and depression symptoms were positively associated with academic burnout. Resilience and self-efficacy were found to mediate the associations partially.

## Background

Academic burnout is a serious and widespread problem that can limit the educational achievement of primary and secondary school students [1, 2]. It is defined as tiredness and loss of interest in learning due to excessive pressure, resulting in emotional exhaustion, depersonalization, and diminished personal accomplishment [3, 4]. Students who experience academic burnout can face considerable mental distress, including feelings of anxiety, depression, frustration, hostility, or fear [4]. As academic burnout increases the likelihood of academic underachievement, truancy, and school dropout, it can seriously impact the learning outcomes and later academic success of affected students [5–7].

One major factor influencing the risk and severity of academic burnout is mental health. In the existing education literature, researchers have repeatedly found that symptoms of mental health conditions like anxiety and depression are correlated with a higher risk of academic burnout across a wide range of student ages [8, 9]. For instance, a cohort study in South Korea found that academic burnout levels among 7th and 8th grade adolescents tended to increase with age and were exacerbated by symptoms of depression [10]. Another study in Spain also identified a correlation between high anxiety levels and high levels of academic burnout among older students who were preparing for university entrance exams [11]. In a study of American physician assistant students, it indicated that anxiety and depression symptoms significantly predicted the emotional exhaustion that is associated with academic burnout, with depression similarly being associated with the depersonalization that is also associated with academic burnout [12].

While academic burnout can arise among students across a wide variety of ages, many researchers have emphasized the importance of studying mental health conditions and academic burnout among students in late primary school and middle school in particular. In addition to exacerbating academic burnout, symptoms of anxiety and depression among students in this age group are associated with a host of negative repercussions, including increased absence rates [13], lower academic achievement [14, 15], and higher risks of emotional exhaustion [12]. Understanding and cultivating protective factors that may mitigate the associations between anxiety or depression symptoms and academic burnout among students is vital.

Resilience and self-efficacy hold promise as protective factors that may prevent or reduce symptoms of anxiety or depression and academic burnout among children and adolescents [1, 16]. Resilience theory suggests that resilient individual has the capability to prevent or overcome negative psychological outcomes, such as anxiety and depression symptoms [17]. Students who exhibit high resilience - the ability to adapt and persevere under pressure or adversity [18] - have been shown to have a relatively greater ability to overcome difficulties, negative emotions, and excessive mental pressure [19, 20]. Studies have demonstrated that resilience is a key mitigating factor for academic burnout, specifically, students with higher level of resilient consistently showed lower level of academic burnout [21, 22]. Self-efficacy, meanwhile, refers to the belief in one’s own ability to perform novel or complex tasks to attain desired outcomes [23]. Theories of self-efficacy posit that different modes of influence alter coping behavior by creating and strengthening expectations of personal efficacy [24]. Individuals with low self-efficacy, on the other hand, may overestimate how challenging problems are, potentially increasing stress, giving rise to depression symptoms, and / or weakening problem-solving abilities [25]. Additionally, low self-efficacy seems related to non-adaptive academic behaviours, which leading to less commitment to school tasks and poor academic performance [26]. Like resilience, research has demonstrated that self-efficacy is effective in protecting students against academic burnout [27].

Studies conducted in China have demonstrated a heightened prevalence of academic burnout in settings characterized by elevated rates of anxiety and depression among children and adolescents. Specifically, approximately 54.9% of secondary school students in China reported experiencing academic burnout [28]. Furthermore, research has revealed that nearly 46% of Chinese students exhibit a lack of interest in learning, 33% display a substantial aversion to studying, and only 21% maintain a truly positive attitude towards the learning process [29]. One plausible explanation for these adverse learning attitudes is rooted in the substantial emphasis placed by China’s educational system on high school and college entrance examinations. The consequent expectations and pressures exerted by parents and educators to excel in these examinations, along with the educational practices leading up to them, can engender considerable academic stress, thereby heightening the susceptibility to academic burnout [30–33]. Moreover, the prevalence of anxiety and depression problems among children and adolescents in China has been rising, especially during the COVID-19 epidemic [34, 35]. Recent studies estimate that 37.4% of Chinese teens exhibit anxiety symptoms, and 19.9 to 43.7% of them exhibit depression symptoms [36, 37].

While the mental health challenges stemming from substantial academic pressure have received substantial attention in research, there is a scarcity of studies in China that investigate the potential mediation effects of resilience and self-efficacy in the associations between anxiety / depression symptoms and academic burnout among primary and middle school students. Mental health symptoms like anxiety and depression correlate with higher academic burnout [38]. Resilience and self-efficacy have been found to be protective in the school context, which increase school engagement and mitigate academic burnout in adolescents [39, 40]. In China, resilience has been shown to moderate the association between adverse life events and mental health problems among students [41]. Resilience may reduce academic burnout, improve emotional regulation, and mediate the association between learning pressures and burnout [42, 43].While Jung et al. [1] found that self-efficacy mediates the relationship between academic stress and academic burnout in China, the specific role of self-efficacy in being able to mitigate the association between anxiety and depression symptoms and academic burnout in China has gone largely unexamined.

**Fig. 1:**
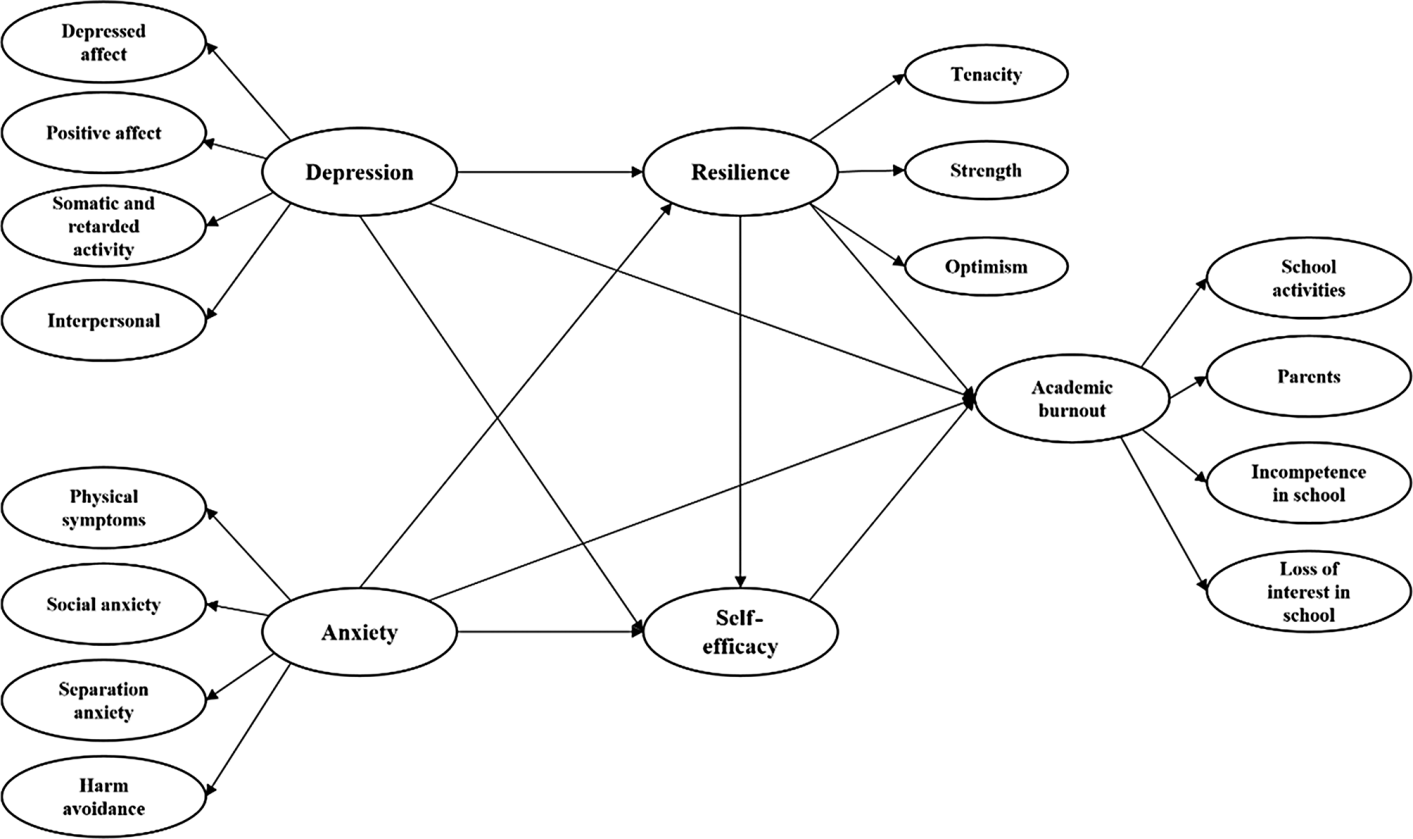
Hypothesized research framework

The current study contributes to the existing literature by investigating the relationships between anxiety, depression symptoms, and academic burnout among primary and middle school students in China, as well as explores the role of resilience and self-efficacy in mitigating academic burnout. To this end, we propose the following specific hypotheses: (1) anxiety and depression symptoms positively correlated with academic burnout; (2) simple medicating role of resilience and self-efficacy between anxiety / depression symptoms and academic burnout; and (3) chain medicating role of resilience and self-efficacy between anxiety / depression symptoms and academic burnout. Fig 1 illustrates the research framework diagram.

## Methods

### Study design

Students were recruited from five schools in Shanghai using a stratified two-stage cluster sampling approach. In the first stage, cluster random sampling was used to select two primary schools and three middle schools in Shanghai. In the second stage, stratified sampling approach was applied to randomly select three classes for each grade from grade 4th to grades 8th. Students were considered eligible for inclusion if they had been studying in the participating school for more than six months, had no known psychiatric diagnoses, and had their parent’s consent to participate. All eligible students in the selected classes were included in the study.

### Participants

A total of 2,070 students aged between 9 and 14 years participated in the surveys. Out of these 2,070 students, 1,105 (53.38%) were boys while 965 (46.62%) were girls. A126 (6.09%) were in the 4th grade, 100 (4.83%) were in the 5th grade, 648 (31.30%) were in the 6th grade, 714 (34.49%) were in the 7th grade, and 482 (23.29%) were in the 8th grade. Regarding family composition, 964 (46.57%) of the students were identified as only children. Family structures varied, with 1,212 (58.55%) in nuclear families, 753 (36.38%) in extended families, and 105 (5.07%) in single-parent families. The highest percentage of students with annual household income is between 15,000 and 30,000 (870 (42.03%)), while 659 (31.84%) students have annual household income less than 15,000, and 541 (26.13%) students have annual family income more than 30,000. Regarding the educational levels of parents, 31.84% of fathers had completed high school, while 36.91% of mothers had attained a lower middle school education. Lastly, a significant portion of parents were under 40 years old, with 52.22% of fathers (1,081) and 64.78% of mothers (1,341) falling into this age category. (Table 1)

**Table 1:** The difference of academic burnout among demographic information

Variables	*N*(%)	Median (p25, p75)	Z/χ^2^
Gender			
Boys	1,105(53.38)	55(43,63)	-0.41
Girls	965(46.62)	55(45,62)	
Grade			
Fourth	126(6.09)	43(39,54)	127.80**
Fifth	100(4.83)	48(39,57)	
Sixth	648(31.30)	53(42,60)	
Seventh	714(34.49)	56(46,65)	
Eighth	482(23.29)	58(51,66)	
One-child family			
Yes	964(46.57)	54.5(43.5,63)	1.28
No	1,106(53.43)	55(44,64)	
Family type			
Nuclear family	1,212(58.55)	55(44,63)	18.60**
Extended family	753(36.38)	55(44,62)	
Single-parent family	105(5.07)	58(53,69)	
Annual household income (USD)			
≤ 15,000	659(31.84)	55(44,64)	7.84*
15,000–30,000	870(42.03)	55.5(45,63)	
>30,000	541(26.13)	54(42,61)	
Father’s education			
Middle school and below	646(31.21)	56(44,64)	11.11*
High school	659(31.84)	55(44,64)	
Junior college	434(20.97)	55(45,62)	
Bachelor or above	331(15.99)	53(42,61)	
Mother’s education			
Middle school and below	764(36.91)	56(44,64)	17.31**
High school	551(26.62)	56(47,64)	
Junior college	467(22.56)	55(44,62)	
Bachelor or above	288(13.91)	53(42,60)	
Father’s age			
≤ 40	1,081(52.22)	54(43,62)	6.81*
40–50	892(43.09)	55(45,64)	
>50	97(4.69)	57(47,65)	
Mother’s age			
≤ 40	1,341(64.78)	54(43,62)	5.46
40–50	675(32.61)	55(45,64)	
>50	54(2.61)	59(49,66)	

Note **p < 0.01, *p < 0.05; Wilcoxon rank sum test statistics Z for two-group comparisons and kruskal-wallis rank sum test statistics χ2 for multiple-group comparisons

### Measures

The study utilized student self-report questionnaires, each assigned a unique identification number that was subsequently anonymized. The specific assessment scales used in this study were as follows:

#### Elementary school student burnout scale for grades 6–8 (ESSBS)

The ESSBS comprises 26 individual items to assess student academic burnout from different possible sources, including school activities (12 items), actions of parents (5 items), incompetence in school (4 items), and loss of interest in school (5 items) [3]. A four-point Likert scale (1 = strongly agree, 2 = agree, 3 = disagree, 4 = strongly disagree) is used, with some items coded reversely. Total possible scores range from 26 to 104, with higher scores reflecting a higher level of academic burnout. The Cronbach’s α for the sub-dimensions range from 0.76 to 0.92 in a previous study [3]. The model indices emerging from the Confirmatory Factor Analysis (Goodness-of-fit Index (GFI) = 0.94, Adjusted Goodness-of-fit Index (AGFI) = 0.91, Parsimony Goodness-of-fit Index (PGFI) = 0.89, Root-mean-square Error of Approximation (RMSEA) = 0.07, Comparative fit Index (CFI) = 0.91) indicate that there was a good fit [3]. In the current study, the Cronbach’s α of the total scale is 0.795. The Cronbach’s α coefficients for each dimension also are relatively high, specifically, 0.902 for the dimension of Burnout Due to School Activities; 0.801 for the dimension of Due to Parents; 0.831 for the dimension of Due to Incompetence in School; and 0.860 for the dimension of Loss of Interest in School.

#### Multidimensional anxiety scale for children-Chinese (MASC-C)

The version of the MASC scale used in China is a 39-item self-reported scale to assess anxiety symptoms experienced by children and adolescents during the week prior to the survey [44]. The items are grouped into the following four dimensions: Physical symptoms (12 items), Social anxiety (9 items), Separation anxiety (9 items), and Harm avoidance (9 items). Each item is rated on a four-point Likert scale (0 = never, 1 = sometimes, 2 = rarely, 3 = always true about me), yielding a total score between 0 and 117. Higher total scores indicate higher levels of anxiety. A previous study indicated that a raw score of 48 was the best cutoff point by ROC (Receiver Operating Characteristic) analysis, which has a high level of sensitivity and specificity [45]. The results of the MASC-C scale showed that the Cronbach’s α for MASC-C was 0.91 and the test-retest reliability was 0.84. Confirmatory factor analysis indicates that there are good fit indices for the factor analysis model with GFI = 0.96, CFI = 0.95, NNFI = 0.94, and RMSEA = 0.06 [44]. For the current sample, the Cronbach’s α is 0.835 for the overall scale of MASC-C, and the Cronbach’s α for the sub-dimensions range from 0.694 to 0.892, indicating relatively high internal consistency.

#### Center for epidemiological studies depression scale (CES-D)

The CES-D is a 20-item self-reported scale used to measure depression symptoms [46]. It contains four sub-dimensions, namely Depressed affect (8 items), Positive affect (4 items), Somatic and retarded activity (6 items), and Interpersonal (2 items). Students are asked to report the frequency of each of the depression symptoms during the week and 3 = most, with positive items scored in reverse). Total scores range from 0 to 60, with scores over 20 points considered indicative of an individual to be at possible risk for depression symptoms [47]. The CES-D scale has high internal consistency reliability, and the factor structure has been verified to be suitable for adolescents in China [48]. In this study, the Cronbach’s α of the overall score is 0.882 and the Cronbach’s α for the sub-dimensions ranged from 0.528 to 0.839.

#### Chinese version of the connor-davidson resilience scale (CD-RISC)

The version of CD-RISC used in China assesses three dimensions of resilience, namely tenacity (13 items), strength (8 items), and optimism (4 items) [49]. Each of the items is rated on a 5-point Likert scale, and the overall score can range from 0 to 125, with higher scores reflecting higher resilience. The Cronbach’s α of China’s version of the scale was 0.91, and the Cronbach’s α for each subscale was 0.88 for Tenacity, 0.80 for Strength, and 0.60 for Optimism [49]. In the present study, the scale shows high internal consistency. The Cronbach’s α is 0.922 for the overall scale of CD-RISC, 0.870 for the Tenacity dimension, 0.836 for the Strength dimension, and 0.536 for the Optimism dimension.

### Chinese version of the general self-efficacy scale (GSES)

The version of the GSES used in China includes 10 items that seek to measure an individual’s self-confidence when met with setbacks or difficulties [50]. Items are rated on a 4-point Likert-type (1 = not at all true, 2 = hardly true, 3 = moderately true, 4 = exactly true). Total scores range from 10 to 40, with higher scores indicating higher levels of self-efficacy. In a previous study based on a sample of 23 nations, the Cronbach’s α of the GSES ranged from 0.76 to 0.90 [50]. The version of the GSES that is used in China has had good reliability and validity in past studies [51]. In this study, the reliability coefficient of the total score is 0.898.

### Data collection

Questionnaires were delivered to the classroom by the head teachers, introduced and elucidated to the participants. Students were required to complete the questionnaire during self-study time. This timing was deliberately chosen to minimize interference with academic activities and to foster a focused environment conducive to thoughtful survey responses. Research staff were tasked with offering precise instructions and overseeing the questionnaire completion process to ensure the questionnaire’s quality. Additionally, they proffered guidance to students as needed during the questionnaire completion, while also ensuring that the completed questionnaires were collected precisely one hour after distribution. The student identification number was used to match student-reported questionnaires.

Before the survey, parental consent was obtained through signed informed consent forms. These files, detailing study objectives, methodologies, and ethical considerations, were thoughtfully prepared and distributed via established channels. Parents meticulously reviewed and signed the informed consent documents, affirming their approval of their children’s participation.

### Statistical analysis

All statistical analyses were conducted using STATA 16.0 and Smart PLS 3.0. Initially, the demographic characteristics of children and adolescents were identified. Due to non-normal data distribution, medians were employed to depict main variable distribution. Wilcoxon and Kruskal-Wallis tests were applied to examine the influence of multiple socio-demographic factors on academic burnout. Subsequently, hierarchical regression analysis investigated associations between anxiety / depression symptoms and academic burnout, and the potential mediating role of resilience or self-efficacy in these associations was explored. Covariates, including grade, gender, one-child family, family type, income, parental education, and parents’ age, were integrated into respective models. The Model 1 only kept the demographic information. The anxiety / depression were added into Model 2, while resilience / self-efficacy were added into Model 3. Statistical significance was set at 0.05 using a two-tailed test.

Finally, the mediating role of resilience and self-efficacy in the relationship between anxiety / depression symptoms and academic burnout was examined using Partial Least Squares Regression Structural Equation Modeling (PLS-SEM). PLS-SEM encompasses a measurement model delineating relationships among latent variables and their indicators, alongside a structural model elucidating interconnections between latent variables. The construct’s reliability and validity were assessed using metrics including average variance extracted (AVE), Cronbach’s alpha, and composite reliability (CR). Discriminant validity was established through the heterotrait-monotrait ratio (HTMT) and correlation [52]. Upon confirming the assumed relationships, statistical significance testing and path coefficient calculations were executed via 5000 resampling bootstraps.

## Results

Table 1 displays the associations between academic burnout and student demographic characteristics. The prevalence of academic burnout in primary and middle school students varied widely by grade, family structure, household income, parental education, and father’s age. Specifically, students in higher grades showed increased levels of academic burnout (χ2 = 127.8, *p* < 0.01). Students in single-parent families had higher levels of academic burnout than those living with nuclear and extended families (χ2 = 18.60, *p* < 0.01), while students with an annual household income greater than $30,000 had lower levels of academic burnout than students with household income between $15,000 - $30,000 and less than $30,000 (χ2 = 7.84, *p* < 0.05). Higher parental education (bachelor’s degree or above) was associated with lower levels of academic burnout (χ2 = 11.11, *p* < 0.05; χ2 = 17.31, *p* < 0.01), and students with older fathers had higher levels of academic burnout (χ2 = 6.81, *p* < 0.05) (see Table 1).

Table 2 presents the results of the hierarchical multiple linear regression analysis of the factors associated with academic burnout in primary and middle school students. The anxiety and depression symptoms in Model 2 were significantly positively correlated with academic burnout (*β* = 0.105, *p* < 0.01; *β* = 0.332, *p* < 0.01). Moreover, in model 3, resilience (*β* = -0.204, *p* < 0.01) and self-efficacy (*β* = -0.186, *p* < 0.01) were significantly negatively associated with academic burnout. Model 2 was better than model 1 (R^2^ = 0.349, ${\Delta }$R^2^ = 0.264) in explaining academic burnout, and model 3 was better than model 2 (R^2^ = 0.446, ${\Delta }$R^2^ = 0.097) (see Table 2).

**Table 2:** The factors associated with academic burnout: hierarchical multiple linear regression

Variables	Model 1	Model 2	Model 3
	β	β	β
Grade(Reference: Fourth)			
Fifth	0.034	0.020	0.045*
Sixth	0.213**	0.141**	0.141**
Seventh	0.351**	0.235**	0.211**
Eighth	0.380**	0.287**	0.261**
Gender(Reference: Boys)			
Girls	-0.016	-0.077**	-0.104**
One-child family(Reference: No)			
Yes	-0.061**	-0.056**	-0.052**
Family type(Reference: Nuclear family)			
Extended family	0.009	0.003	-0.001
Single-parent family	0.099**	0.057**	0.033
Family annual income (USD)(Reference: ≤15,000)			
15,000–30,000	0.002	-0.001	-0.000
>30,000	-0.016	-0.028	-0.025
Father’s education(Reference: Middle school and below)			
High school	-0.012	-0.031	-0.020
Junior college	0.002	-0.002	0.002
Bachelor or above	-0.056	-0.038	-0.018
Mother’s education(Reference: Junior high and below)			
High school	0.028	0.028	0.037
Junior college	-0.027	0.005	0.025
Bachelor or above	-0.052	-0.022	0.002
Father’s age(Reference: ≤40)			
40–50	0.001	0.006	0.011
>50	0.009	-0.009	-0.017
Anxiety symptoms		0.134**	0.105**
Depression symptoms		0.434**	0.332**
Resilience			-0.204**
Self-efficacy			-0.186**
Constant	47.301**	41.476**	65.579**
R^2^	0.085	0.349	0.446
${\Delta }$ R^2^	0.085	0.264	0.097

Note **p < 0.01, *p < 0.05. All parameters of *β* are standardized

Table 3 accesses the model’s construct reliability and validity were evaluated. Average variance extracted (AVE) > 0.50, heterotrait-monotrait ratio (HTMT) < 0.85, Cronbach’s alpha > 0.70, and composite reliability (CR) > 0.80 were used to assess the convergent validity, discriminant validity, and composite reliability of the reflective measurement model (see Table 3). It was tested that there was no multicollinearity between variables of this study. The structural equation modeling R^2^ with academic burnout as the dependent variable was 0.432 as obtained from model fitting, indicating a moderate degree of explanation. By Blindfold method validation, the obtained Q^2^ = 0.289 (Q^2^ > 0), so this model can be considered reliable. GoF is an indicator of the goodness of fit of the PLS-SEM model, calculated so that the model’s goodness of fit can be considered moderate (Gof = 0.323). We verified the reasonableness of the assumed relationship in the model.

**Table 3:** Reflective measurement model validity and reliability analysis among latent variables

	Convergent validity	HTMT					Reliability	
	AVE	Academic burnout	Resilience	Depression symptoms	Anxiety symptoms	Self-efficacy	Cronbach’s alpha	CR
Academic burnout	0.675						0.836	0.892
Resilience	0.777	0.549					0.856	0.912
Depression symptoms	0.800	0.629	0.404				0.916	0.941
Anxiety symptoms	0.677	0.475	0.289	0.664			0.841	0.893
Self-efficacy	0.527	0.534	0.649	0.331	0.295		0.887	0.907

*Note* AVE average variance extracted; HTMT heterotrait-monotrait ratio; CR composite reliability. In the inner model, AVE > 0.50, HTMT < 0.85, Cronbach’s alpha > 0.70, and CR > 0.80 indicate good convergent validity, discriminant validity, and reliability

**Fig. 2:**
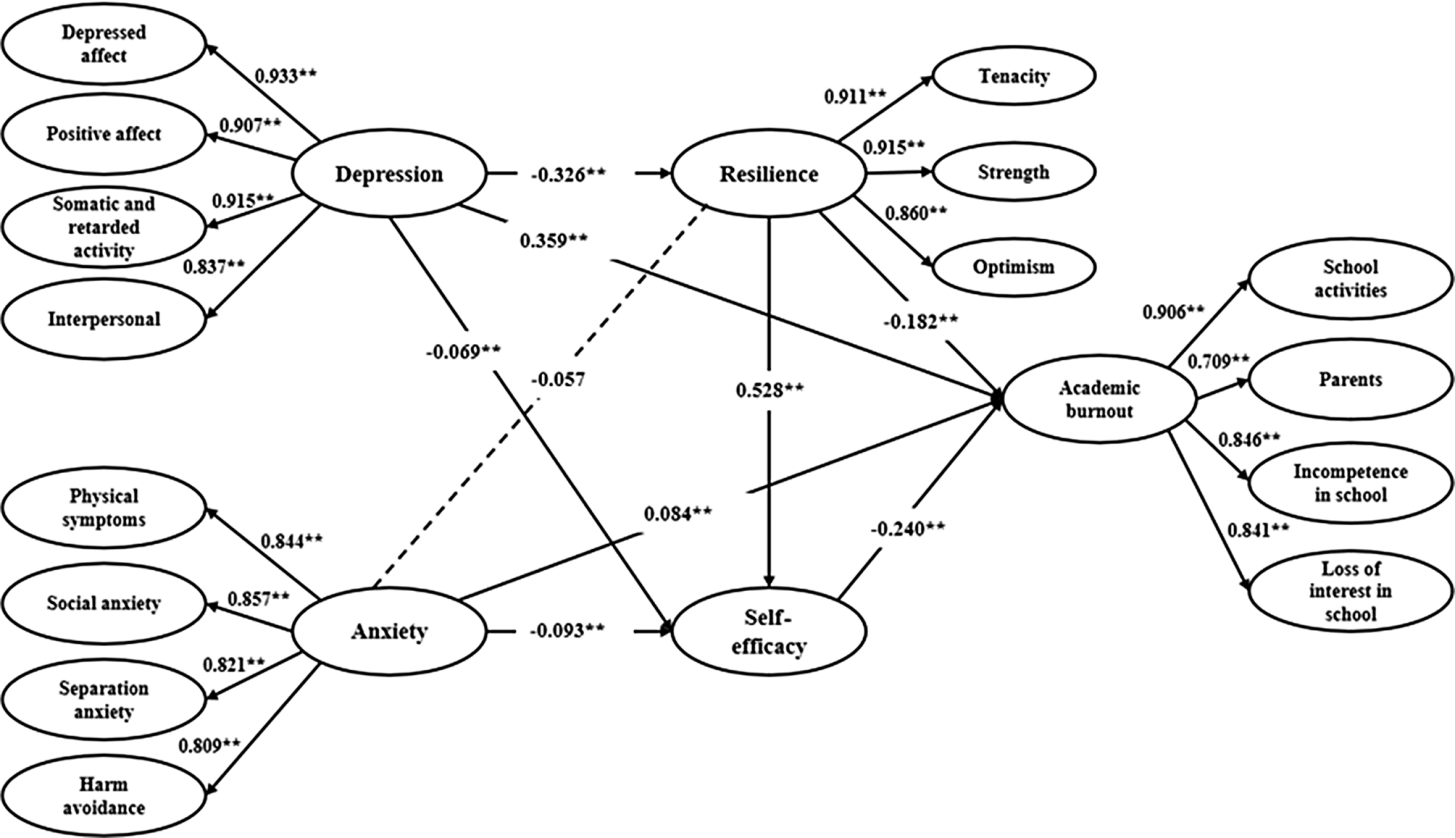
The SEM model for the associations between anxiety symptoms, depression symptoms, and Academic Burnout Note **p < 0.01. All parameters of β are standardized

Table 4 and Fig. 2 show the path from anxiety and depression symptoms to academic burnout. Anxiety symptoms are positively correlated with academic burnout (*β* = 0.124, *p* < 0.01) and negatively correlated with self-efficacy, and self-efficacy partially mediates the association between anxiety symptoms and academic burnout (*β* = 0.022, *p* < 0.01). In other words, self-efficacy mediates 17.74% of the association between anxiety symptoms and academic burnout. No significant correlation is found between anxiety symptoms and resilience, and resilience does not significantly mediate the association between anxiety symptoms and academic burnout. Similarly, depression symptoms are positively associated with academic burnout (*β* = 0.477, *p* < 0.01) and negatively correlated with self-efficacy, with self-efficacy mediating 3.56% of the association between depression symptoms and academic burnout (*β* = 0.017, *p* < 0.01). Depression symptoms are also negatively correlated with resilience, and resilience partially mediates 12.37% of the association between anxiety symptoms and academic burnout (*β* = 0.059, *p* < 0.01). Additionally, resilience and self-efficacy together mediate 8.60% of the association between depression symptoms and academic burnout (*β* = 0.041, *p* < 0.01).

**Table 4:** Path coefficients among structural equation modeling

Path of latent variables	Bootstrapping		
	β	95%CI	t
**Direct effect**			
Anxiety symptoms → Academic burnout	0.124**	(0.077, 0.173)	5.027
Depression symptoms →Academic burnout	0.477**	(0.432, 0.519)	21.454
Resilience → Academic burnout	-0.309**	(-0.347, -0.269)	15.42
Self-efficacy → Academic burnout	-0.24**	(-0.281, -0.197)	11.251
**Indirect effect**			
Anxiety symptoms → Resilience → Academic burnout	0.01	(0, 0.022)	1.822
Anxiety symptoms → Self-efficacy → Academic burnout	0.022**	(0.011, 0.035)	3.67
Depression symptoms → Resilience → Academic burnout	0.059**	(0.043, 0.079)	6.578
Depression symptoms → Self-efficacy → Academic burnout	0.017**	(0.005, 0.029)	2.666
Resilience → Self-efficacy → Academic burnout	-0.127**	(-0.152, -0.104)	10.249
Anxiety symptoms → Resilience → Self-efficacy → Academic burnout	0.007	(0, 0.015)	1.833
Depression symptoms → Resilience → Self-efficacy → Academic burnout	0.041**	(0.031, 0.054)	7.331

Note **p < 0.01. All parameters of β are standardized

## Discussion

The current study offers evidence that anxiety and depression symptoms are associated with a higher risk of academic burnout. Our findings related to anxiety are consistent with several past studies showing that students with higher levels of anxiety experience higher levels of academic burnout [9, 11]. The literature also provides several reasons that anxiety might contribute to higher levels of burnout. First, high levels of stress, an important correlate of anxiety, can increase the risk of emotional exhaustion, a major component of academic burnout [12, 53]. Second, according to the Stress-Alexithymia Hypothesis [54], experiencing academic stress for prolonged periods is often associated with difficulties in confronting and regulating emotions, which has been shown to lead to harmful consequences like burnout, including academic burnout [54, 55]. Finally, prolonged stress related to fears of academic failure or criticism over academic performance may increase the likelihood that students develop a negative self-image, which has been found to decrease one’s impulse control and one’s capacity to deal with the demands of the external world, thereby increasing the risk of emotional exhaustion and burnout [56].

The present study also verified the findings of several previous studies that depression symptoms are associated with academic burnout, with more severe symptoms correlating to higher levels of academic burnout [10, 57, 58]. Several factors may help explain this association. Higher expectations from parents regarding their children’s academic performance resulted in elevated levels of academic stress and depression among students, especially rooted in China’s educational system [33, 59, 60]. Research in adults shows that depression symptoms, negative emotions, and sensitivity to the outside world predispose people to burnout [61]. People living with depression also often perceive and evaluate their experiences more negatively than those who are not depressed [8]. Students with more serious symptoms of depression may produce more negative attitudes toward learning, which may lead to emotional exhaustion, a reduced sense of achievement, and higher levels of academic burnout. In this way, depression symptoms and academic burnout may feed into each other, forming a vicious cycle [8].

The analysis in the paper demonstrates that self-efficacy is not only negatively associated with academic burnout, but also partially mediates the association between anxiety / depression symptoms and academic burnout. These findings are consistent with previous studies that have associated low self-efficacy with higher levels of academic burnout [1, 25, 62] and shown its mediator role between external stress and burnout [1, 63]. A likely explanation for self-efficacy’s role in mitigating the association between anxiety symptoms and academic burnout can be found in social cognitive theory, which models how self-efficacy critically influences one’s perception of potential challenges. Individuals with low self-efficacy do not believe they can manage threatening events and are more likely to feel apprehensive in their daily lives, which is what produces higher levels of anxiety [64]. Individuals with high self-efficacy, on the other hand, feel less overwhelmed by potential challenges. Therefore, even when facing academic stress (a correlate of anxiety), they tend to be less vulnerable to academic burnout [1]. Self-efficacy mediates the association between depression symptoms and academic burnout in a similar way. Students who experience depression symptoms and have low self-efficacy tend to have more trouble processing events, which can contribute to emotional exhaustion, a major component of academic burnout [65, 66]. In contrast, even when facing symptoms of depression, students with higher self-efficacy may be more able to adapt to life changes, keep calm when facing difficult assignments and activities, and select appropriate study strategies to face academic assignments, thereby better preventing academic burnout.

We find that resilience, like self-efficacy, partially mediates the association between symptoms of depression and academic burnout. This result echoes prior studies that have found that resilience acts as a multiple mediator in the association between emotional problems and other mental health problems, including academic burnout [67]. Specifically, even when depressed, students with high resilience were able to manage stress and learn from stressful experiences, while those with low resilience found recovering from academic stress and exhaustion more difficult [16]. While this prior study focused on older medical students, a similar buffering effect likely explains the mediating role of resilience between depression and academic burnout among children and adolescent students that was found in the results of the current study.

### Chain mediating role of resilience and self-efficacy

A major contribution of this study is our documentation of the partial chain mediating role of resilience and self-efficacy between depression symptoms and academic burnout. We found that resilience was positively associated with self-efficacy, which is consistent with past findings that self-efficacy is closely linked to components of resilience including positive relationships with caring adults, strong problem-solving skills, and strong intellectual functioning [68]. Individuals that have high self-efficacy believe that they have the capacity to influence the outcome of events in their own lives, which can also contribute to the development of competency in the face of adversity, an important aspect of resilience [69]. Similarly, students with higher resilience have been found to perceive themselves as more efficient both in general and in academic contexts—in other words, to have higher self-efficacy [68]. This association between resilience and self-efficacy means that children and adolescents may have higher self-efficacy when facing new external pressures if they also have strong resilience, and vice versa. Self-efficacy and resilience support each other, helping students in which both self-efficacy and resilience are present to better cope with negative emotions and to lower the risk of academic burnout from negative emotional states such as depression.

This study, however, did not find that resilience mediates the association between anxiety and academic burnout, nor did it find that resilience and self-efficacy chain-mediate between anxiety symptoms and academic burnout. This finding may be because the negative association between anxiety symptoms and resilience was not significant in the current study. Given that resilience was negatively correlated with depression, we would have expected it to be negatively associated with anxiety as well, an expectation that is supported by previous studies. An earlier study in Ghana, for instance, found that resilience was negatively correlated with anxiety and partially explained the variance between stress and anxiety symptoms [70]. The lack of a similar finding in this study has two possible explanations. First, the children and adolescents in China who received high resilience scores in this study may have been resilient to some kinds of outcomes but not others, such as anxiety. Secondly, resilience may be context-dependent, meaning that resilient individuals in some contexts or age groups may cope better with anxiety than others. The mechanism of the association between anxiety symptoms and resilience remains an important question that should be explored in further studies.

### Limitations

Several additional limitations in the present study merit mention. First, as the present study uses cross-sectional data, it lacks temporal evidence for causality. Future studies should use longitudinal designs to explore the causal relationships between mental health, psychological traits, and academic burnout. Second, the data were collected by self-reported questionnaires, which can be influenced by social desirability bias and recall bias. Third, the present study was conducted in primary and middle school students from a specific region in China, potentially limiting the generalizability of our findings.

Despite these limitations, this study provides new evidence about the associations between anxiety and depression symptoms and academic burnout, as well as the important role played by self-efficacy and resilience in mitigating these associations. The findings can be used to guide future interventions aimed at promoting mental health and guarding against academic burnout among students in China and abroad.

## Conclusions

The current study explores the associations between anxiety and depression symptoms and academic burnout among students in China, and examines the role of resilience and self-efficacy in addressing academic burnout. We found that anxiety and depression symptoms are positively associated with academic burnout. Furthermore, resilience and self-efficacy hold promise as protective factors that can partially mediate the positive associations. These findings would suggest that interventions targeting resilience and self-efficacy may be a preventive measure against negative emotions, and mitigate academic burnout. In this respect, by strengthening home-school links, parents and teachers should encourage students to foster a growth mindset bounce back from adversities, and promote positive thinking to help students positively cope with life’s stresses and challenges. Additionally, school-based educators and relevant authorities should conduct regular mental health monitoring and evaluation, which can identify anxiety and depression levels promptly to mitigate the onset of academic burnout.

## Data Availability

The data are available from the corresponding author upon reasonable request.

## Abbreviations

AGFI: Adjusted Goodness-of-Fit Index
AVE: Average Variance Extracted
CD-RISC: Connor-Davidson Resilience Scale
CES-D: Center for Epidemiological Studies Depression Scale
CFI: Comparative Fit Index
CR: Composite Reliability
ESSBS: Elementary School Student Burnout Scale
GFI: Goodness-of-Fit Index
GSES: General Self-Efficacy Scale
HTMT: Heterotrait-Monotrait Ratio
MASC-C: Multidimensional Anxiety Scale for Children-Chinese
PGFI: Parsimony Goodness-of-Fit Index
PLS-SEM: Partial Least Squares Regression Structural Equation Modeling
RMSEA: Root-Mean-Square Error of Approximation
